# Crystal structure of 2-methyl-4-[(thio­phen-2-yl)methyl­idene]-1,3-oxazol-5(4*H*)-one

**DOI:** 10.1107/S2056989015000833

**Published:** 2015-01-21

**Authors:** Preetika Sharma, K. N. Subbulakshmi, B. Narayana, K. Byrappa, Rajni Kant

**Affiliations:** a Post-Graduate Department of Physics & Electronics, University of Jammu, Jammu Tawi 180 006, India; b Department of Chemistry, Mangalore University, Mangalagangotri 574 199, D. K., Mangalore, India; c Mangalore University, Mansagangotri, Mangalore, India

**Keywords:** crystal structure, azlactones, 1,3-oxazol-5(4*H*)-one, hydrogen bonding, C—H⋯π and π–π inter­actions

## Abstract

The asymmetric unit of the title compound, C_9_H_7_NO_2_S, contains two crystallographically independent mol­ecules (*A* and *B*). Both mol­ecules are almost planar [maximum deviations = 0.047 (1) and 0.090 (1) Å, respectively, for the S atoms] with the oxazole and thio­phene rings being inclined to one another by 2.65 (16)° in mol­ecule *A* and by 4.55 (15)° in mol­ecule *B*. In the crystal, the individual mol­ecules are linked *via* C—H⋯O hydrogen bonds, forming –*A*–*B*–*A*–*B*– chains along the [10-1] direction. The chains are linked *via* C—H⋯π and π–π inter­actions [inter­centroid distances = 3.767 (2) and 3.867 (2) Å] involving inversion-related oxazole and thio­phene rings in both mol­ecules, forming a three-dimensional structure.

## Related literature   

For the different roles of 1,3-oxazol-5(4*H*)-one derivatives, see: Etschenberg *et al.* (1980 ▸); Reed & Kingston (1986 ▸). For the crystal structure of 2-(naphthalen-1-yl)-4-[(thio­phen-2-yl)methyl­idene]-1,3-oxazol-5(4*H*)-one, see: Gündoğdu *et al.* (2011*b* ▸). For the crystal structures of some oxazole compounds, see: Gündoğdu *et al.* (2011*a* ▸); Sun & Cui (2008 ▸); Huang *et al.* (2012 ▸); Asiri & Ng (2009 ▸).
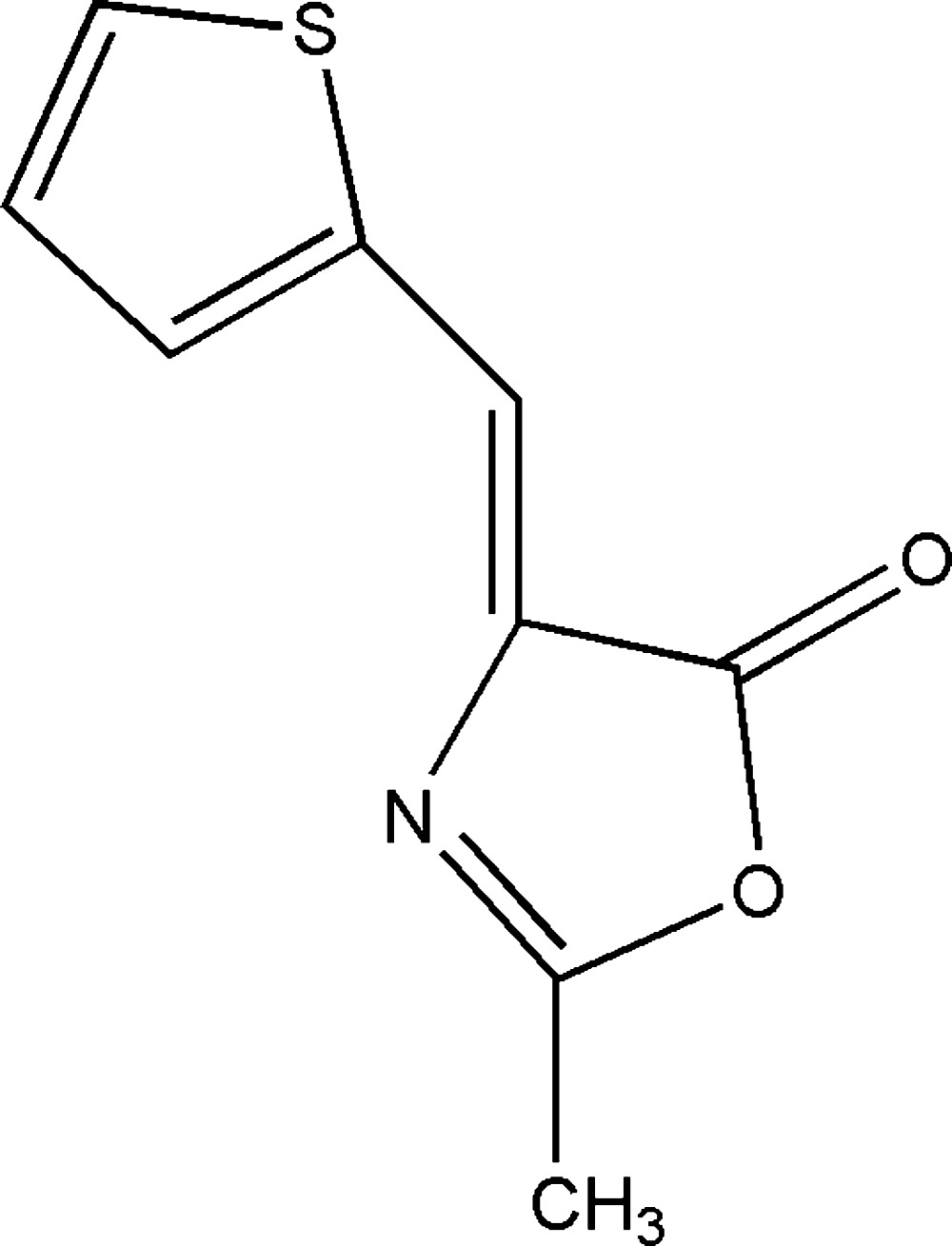


## Experimental   

### Crystal data  

C_9_H_7_NO_2_S
*M* *_r_* = 193.22
Monoclinic, 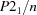

*a* = 12.2264 (11) Å
*b* = 9.8581 (7) Å
*c* = 15.8735 (13) Å
β = 112.129 (10)°
*V* = 1772.3 (2) Å^3^
*Z* = 8
Mo *K*α radiation
μ = 0.33 mm^−1^
*T* = 293 K
0.30 × 0.20 × 0.20 mm

### Data collection  

Oxford Diffraction Xcalibur Sapphire3 diffractometer
Absorption correction: multi-scan (*CrysAlis RED*; Oxford Diffraction, 2010 ▸) *T*_min_ = 0.842, *T*_max_ = 1.000
7052 measured reflections
3472 independent reflections
2477 reflections with *I* > 2σ(*I*)
*R*_int_ = 0.029

### Refinement  

*R*[*F* ^2^ > 2σ(*F* ^2^)] = 0.045
*wR*(*F* ^2^) = 0.126
*S* = 1.04
3472 reflections
237 parameters
H-atom parameters constrained
Δρ_max_ = 0.23 e Å^−3^
Δρ_min_ = −0.34 e Å^−3^

### 

Data collection: *CrysAlis PRO* (Oxford Diffraction, 2010 ▸); cell refinement: *CrysAlis PRO*; data reduction: *CrysAlis PRO*; program(s) used to solve structure: *SHELXS97* (Sheldrick, 2008 ▸); program(s) used to refine structure: *SHELXL97* (Sheldrick, 2015 ▸); molecular graphics: *ORTEP-3 for Windows* (Farrugia, 2012 ▸); software used to prepare material for publication: *PLATON* (Spek, 2009 ▸).

## Supplementary Material

Crystal structure: contains datablock(s) I, New_Global_Publ_Block. DOI: 10.1107/S2056989015000833/su5054sup1.cif

Structure factors: contains datablock(s) I. DOI: 10.1107/S2056989015000833/su5054Isup2.hkl

Supporting information file. DOI: 10.1107/S2056989015000833/su5054Isup3.cml

**Fig. 1.**
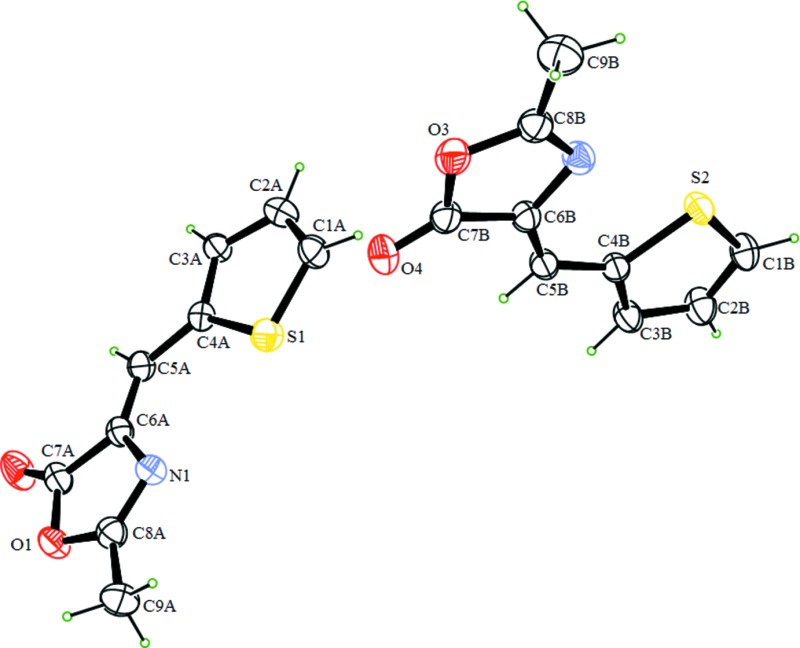
A view of the molecular structure of the two independent molecules of the title compound, with atom labelling. Displacement ellipsoids are drawn at the 40% probability level.

**Fig. 2.**
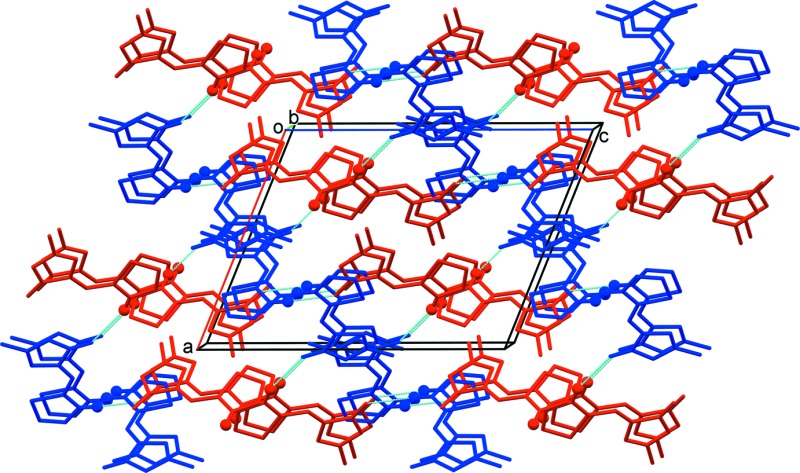
A view along the b axis of the crystal packing of the title compound. The C—H···O ydrogen bonds are shown as dashed lines (see Table 1 for details; molecule A blue; molecule B red).

. DOI: 10.1107/S2056989015000833/su5054fig1.tif
A view of the mol­ecular structure of the two independent mol­ecules of the title compound, with atom labelling. Displacement ellipsoids are drawn at the 40% probability level.

b A B . DOI: 10.1107/S2056989015000833/su5054fig2.tif
A view along the *b* axis of the crystal packing of the title compound. The C—H⋯O ydrogen bonds are shown as dashed lines (see Table 1 for details; mol­ecule *A* blue; mol­ecule *B* red).

CCDC reference: 1043723

Additional supporting information:  crystallographic information; 3D view; checkCIF report

## Figures and Tables

**Table 1:** Hydrogen-bond geometry (, ) *Cg*1 is the centroid of the thiophene ring S1*A*/C1*A*C4*A*.

*D*H*A*	*D*H	H*A*	*D* *A*	*D*H*A*
C3*A*H3*A*O2*B* ^i^	0.93	2.56	3.449(3)	161
C3*B*H3*B*O2*A* ^ii^	0.93	2.49	3.336(3)	151
C9*B*H9*B*2*Cg*1^iii^	0.96	2.96	3.783(4)	145

Symmetry codes: (i) 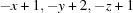; (ii) 
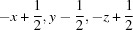
; (iii) 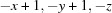.

## supplementary crystallographic information

### S1. Comment

Erlenmeyer azlactones have been used in a wide variety of reactions as precursors for biologically active peptides (Etschenberg *et al.*, 1980; Reed & Kingston, 1986), herbicides and fungicides, pesticides, agrochemical intermediates and as drugs. The crystal structures of some 1,3-oxazol-5(4*H*)-one derivative *viz*., 2-(naphthalen-1-yl)-4-(naphthalen-1-ylmethylidene)-1,3-oxazol-5(4*H*)-one (Gündoğdu *et al.*, 2011a), 2-phenyl-4-(3,4,5-trimethoxybenzylidene)-1,3-oxazol-5(4*H*)-one (Sun & Cui, 2008), 4-[(3-methoxyanilino)methylidene]-2-phenyl-1,3-oxazol-5(4*H*)-one (Huang *et al.*, 2012), (*E*)-4-(2,5-dimethoxybenzylidene)-2-phenyl-1,3-oxazol-5(4*H*)-one (Asiri & Ng, 2009) have been reported. In view of the importance of 1,3-oxazol-5(4*H*)-one, we report herein on the crystal structure of the title compound.

The asymmetric unit of the title compound, Fig. 1, contains two crystallographically independent molecules (A and B), which are almost identical (Fig. 2). The molecular structure is comprised of an oxazole and a thiophene ring which are almost coplanar with a dihedral angle between the rings of 2.65 (16)° in molecule A and 4.55 (15)° in molecule B. All the bond lengths and angles of the title molecule are within normal ranges, and are close to those observed for a very similar structure, viz. 2-(naphthalen-1-yl)-4-[(thiophen-2-yl)methylidene]-1,3-oxazol-5(4*H*)-one (Gündoğdu *et al.*, 2011b).

In the crystal, the individual molecules are linked via C—H···O hydrogen bonds forming -A-B-A-B- chains along direction [101]; Fig. 2 and Table 1. The chains are linked via C-H···π (Table 1) and π-π interactions forming a three dimensional structure [Cg1···Cg2^i^ = 3.767 (2) A° and Cg3···Cg4^ii^ = 3.886 (2) Å where Cg1, Cg2, Cg3 and Cg4 are the centroids of rings S1A/C1A-C4A, O1A/N1A/C6A-C8A, S1B/C1B-C4B and O1B/N1B/C6B-C8B, respectively, with symmetry codes: (i) -x+1, -y+1, -z and (ii) -x+1, -y+1, -z+1].

### S2. Experimental

A mixture of acetyl glycine (2 g, 0.017 mol), thiophene-2-carbaldehyde (1.91 g, 0.017 mol), anhydrous sodium acetate (1.39 g, 0.017 mol) and acetic anhydride (5.20 g, 0.051 mol) was heated on electric plate with constant stirring. As soon as the mixture liquefied completely, the resulting solution was refluxed for 2 h. 25 ml of ethanol was added slowly to the contents of the flask and the mixture was allowed to stand overnight in a refrigerator. The solid mass that separated out was stirred with 60 ml of cold water, filtered, washed with cold water and recrystallized from carbon tetrachloride. Single crystals were grown from chloroform by the slow evaporation method (m.p.: 411-412 K).

### S3. Refinement

All the H atoms were positioned geometrically and refined using a riding model: C—H = 0.93–0.96 Å with *U*_iso_(H) = 1.5*U*_eq_(C) for methyl H atoms and = 1.2*U*_eq_(C) for other H atoms.

### Crystal data

**Table:**

C_9_H_7_NO_2_S	*F*(000) = 800
*M**_r_* = 193.22	*D*_x_ = 1.448 Mg m^−^^3^
Monoclinic, *P*2_1_/*n*	Mo *K*α radiation, λ = 0.71073 Å
Hall symbol: -P 2yn	Cell parameters from 2325 reflections
*a* = 12.2264 (11) Å	θ = 4.2–29.2°
*b* = 9.8581 (7) Å	µ = 0.33 mm^−^^1^
*c* = 15.8735 (13) Å	*T* = 293 K
β = 112.129 (10)°	Block, white
*V* = 1772.3 (2) Å^3^	0.30 × 0.20 × 0.20 mm
*Z* = 8	

### Data collection

**Table:**

Oxford Diffraction Xcalibur Sapphire3 diffractometer	3472 independent reflections
Radiation source: fine-focus sealed tube	2477 reflections with *I* > 2σ(*I*)
Graphite monochromator	*R*_int_ = 0.029
ω scans	θ_max_ = 26.0°, θ_min_ = 3.9°
Absorption correction: multi-scan (*CrysAlis RED*; Oxford Diffraction, 2010)	*h* = −15→9
*T*_min_ = 0.842, *T*_max_ = 1.000	*k* = −12→10
7052 measured reflections	*l* = −19→18

### Refinement

**Table:**

Refinement on *F*^2^	Primary atom site location: structure-invariant direct methods
Least-squares matrix: full	Secondary atom site location: difference Fourier map
*R*[*F*^2^ > 2σ(*F*^2^)] = 0.045	Hydrogen site location: inferred from neighbouring sites
*wR*(*F*^2^) = 0.126	H-atom parameters constrained
*S* = 1.03	*w* = 1/[σ^2^(*F*_o_^2^) + (0.0531*P*)^2^ + 0.4552*P*] where *P* = (*F*_o_^2^ + 2*F*_c_^2^)/3
3472 reflections	(Δ/σ)_max_ = 0.001
237 parameters	Δρ_max_ = 0.23 e Å^−^^3^
0 restraints	Δρ_min_ = −0.34 e Å^−^^3^

### Special details

**Table:**

Experimental. *CrysAlis PRO*, Agilent Technologies, Version 1.171.36.28 (release 01–02-2013 CrysAlis171. NET) (compiled Feb 1 2013,16:14:44) Empirical absorption correction using spherical harmonics, implemented in SCALE3 ABSPACK scaling algorithm.
Geometry. All e.s.d.'s (except the e.s.d. in the dihedral angle between two l.s. planes) are estimated using the full covariance matrix. The cell e.s.d.'s are taken into account individually in the estimation of e.s.d.'s in distances, angles and torsion angles; correlations between e.s.d.'s in cell parameters are only used when they are defined by crystal symmetry. An approximate (isotropic) treatment of cell e.s.d.'s is used for estimating e.s.d.'s involving l.s. planes.
Refinement. Refinement of *F*^2^ against ALL reflections. The weighted *R*-factor *wR* and goodness of fit *S* are based on *F*^2^, conventional *R*-factors *R* are based on *F*, with *F* set to zero for negative *F*^2^. The threshold expression of *F*^2^ > σ(*F*^2^) is used only for calculating *R*-factors(gt) *etc*. and is not relevant to the choice of reflections for refinement. *R*-factors based on *F*^2^ are statistically about twice as large as those based on *F*, and *R*- factors based on ALL data will be even larger.

### Fractional atomic coordinates and isotropic or equivalent isotropic displacement parameters (Å^2^)

**Table:**

	*x*	*y*	*z*	*U*_iso_*/*U*_eq_	
S1A	0.74841 (6)	0.94228 (7)	0.01475 (4)	0.0500 (2)	
S1B	0.40438 (6)	1.07198 (8)	0.32806 (5)	0.0541 (2)	
O1B	0.38555 (17)	1.22165 (19)	0.63729 (11)	0.0545 (5)	
O1A	0.45883 (16)	1.33631 (19)	0.00039 (13)	0.0563 (5)	
N1A	0.57961 (19)	1.1832 (2)	−0.02547 (14)	0.0480 (5)	
N1B	0.42087 (19)	1.2003 (2)	0.50804 (14)	0.0458 (5)	
C5B	0.2829 (2)	1.0126 (3)	0.44127 (16)	0.0433 (6)	
H5B	0.2275	0.9572	0.4513	0.052*	
O2B	0.2489 (2)	1.0587 (2)	0.61918 (14)	0.0730 (7)	
C4A	0.7190 (2)	0.9654 (3)	0.11220 (16)	0.0415 (6)	
O2A	0.47256 (17)	1.2787 (2)	0.14163 (13)	0.0672 (6)	
C5A	0.6410 (2)	1.0659 (3)	0.12201 (17)	0.0445 (6)	
H5A	0.6315	1.0662	0.1774	0.053*	
C6A	0.5796 (2)	1.1602 (3)	0.06201 (17)	0.0433 (6)	
C4B	0.3004 (2)	0.9890 (2)	0.35782 (16)	0.0403 (6)	
C7A	0.5013 (2)	1.2586 (3)	0.07877 (19)	0.0500 (7)	
C3B	0.2367 (2)	0.8952 (3)	0.29012 (16)	0.0442 (6)	
H3B	0.1768	0.8396	0.2930	0.053*	
C8A	0.5108 (2)	1.2838 (3)	−0.05642 (18)	0.0504 (7)	
C3A	0.7808 (2)	0.8734 (3)	0.17737 (17)	0.0517 (7)	
H3A	0.7770	0.8694	0.2347	0.062*	
C6B	0.3358 (2)	1.1033 (3)	0.50605 (16)	0.0430 (6)	
C7B	0.3128 (2)	1.1171 (3)	0.59019 (18)	0.0511 (7)	
C8B	0.4457 (2)	1.2636 (3)	0.58308 (18)	0.0496 (7)	
C2A	0.8503 (3)	0.7863 (3)	0.1489 (2)	0.0606 (8)	
H2A	0.8977	0.7184	0.1852	0.073*	
C9B	0.5305 (3)	1.3745 (3)	0.6211 (2)	0.0647 (8)	
H9B1	0.5995	1.3399	0.6689	0.097*	
H9B2	0.4949	1.4435	0.6450	0.097*	
H9B3	0.5526	1.4125	0.5741	0.097*	
C1B	0.3648 (3)	0.9860 (3)	0.22912 (18)	0.0571 (7)	
H1B	0.4004	0.9985	0.1872	0.068*	
C2B	0.2765 (3)	0.8976 (3)	0.21714 (18)	0.0581 (7)	
H2B	0.2446	0.8432	0.1658	0.070*	
C1A	0.8410 (3)	0.8114 (3)	0.06350 (19)	0.0560 (7)	
H1A	0.8810	0.7624	0.0340	0.067*	
C9A	0.4771 (3)	1.3503 (3)	−0.1459 (2)	0.0706 (9)	
H9A1	0.5186	1.3084	−0.1798	0.106*	
H9A2	0.4972	1.4448	−0.1375	0.106*	
H9A3	0.3936	1.3408	−0.1787	0.106*	

### Atomic displacement parameters (Å^2^)

**Table:**

	*U*^11^	*U*^22^	*U*^33^	*U*^12^	*U*^13^	*U*^23^
S1A	0.0587 (4)	0.0545 (4)	0.0420 (4)	0.0065 (3)	0.0249 (3)	−0.0013 (3)
S1B	0.0542 (4)	0.0596 (5)	0.0523 (4)	−0.0088 (4)	0.0244 (3)	0.0029 (3)
O1B	0.0650 (12)	0.0575 (12)	0.0457 (10)	−0.0038 (10)	0.0261 (9)	−0.0097 (9)
O1A	0.0525 (11)	0.0511 (11)	0.0619 (12)	0.0123 (10)	0.0176 (9)	−0.0031 (10)
N1A	0.0516 (13)	0.0454 (13)	0.0478 (12)	0.0037 (11)	0.0197 (10)	−0.0024 (11)
N1B	0.0505 (13)	0.0448 (12)	0.0431 (12)	−0.0026 (11)	0.0187 (10)	−0.0015 (10)
C5B	0.0453 (14)	0.0428 (14)	0.0458 (14)	−0.0010 (13)	0.0217 (12)	0.0025 (13)
O2B	0.0929 (17)	0.0800 (15)	0.0692 (13)	−0.0172 (13)	0.0566 (13)	−0.0081 (11)
C4A	0.0420 (13)	0.0436 (14)	0.0419 (13)	−0.0010 (12)	0.0190 (11)	−0.0035 (12)
O2A	0.0638 (13)	0.0782 (14)	0.0669 (13)	0.0154 (11)	0.0327 (11)	−0.0119 (11)
C5A	0.0471 (15)	0.0476 (16)	0.0429 (14)	0.0002 (13)	0.0217 (12)	−0.0041 (13)
C6A	0.0416 (14)	0.0434 (15)	0.0461 (14)	0.0008 (12)	0.0178 (11)	−0.0062 (13)
C4B	0.0417 (13)	0.0380 (14)	0.0439 (13)	0.0027 (12)	0.0191 (11)	0.0049 (12)
C7A	0.0422 (15)	0.0516 (17)	0.0550 (16)	0.0030 (13)	0.0169 (13)	−0.0084 (14)
C3B	0.0474 (15)	0.0451 (15)	0.0429 (14)	−0.0044 (13)	0.0201 (12)	−0.0036 (12)
C8A	0.0478 (15)	0.0485 (16)	0.0525 (16)	−0.0018 (14)	0.0160 (13)	−0.0062 (14)
C3A	0.0594 (17)	0.0580 (17)	0.0428 (14)	0.0134 (15)	0.0252 (13)	0.0071 (14)
C6B	0.0462 (14)	0.0445 (14)	0.0421 (13)	0.0019 (13)	0.0209 (11)	0.0018 (12)
C7B	0.0584 (17)	0.0526 (17)	0.0474 (15)	0.0049 (15)	0.0258 (14)	−0.0014 (14)
C8B	0.0521 (16)	0.0468 (16)	0.0496 (15)	0.0044 (13)	0.0189 (13)	0.0014 (13)
C2A	0.0675 (19)	0.0581 (18)	0.0624 (18)	0.0195 (16)	0.0317 (16)	0.0121 (15)
C9B	0.0684 (19)	0.0578 (19)	0.0632 (18)	−0.0108 (17)	0.0194 (16)	−0.0138 (16)
C1B	0.0615 (18)	0.071 (2)	0.0457 (15)	0.0025 (17)	0.0287 (14)	0.0065 (15)
C2B	0.0682 (19)	0.0596 (18)	0.0437 (15)	−0.0025 (16)	0.0181 (14)	−0.0075 (14)
C1A	0.0621 (17)	0.0513 (17)	0.0645 (18)	0.0108 (15)	0.0350 (15)	−0.0046 (15)
C9A	0.081 (2)	0.064 (2)	0.0627 (19)	0.0044 (18)	0.0216 (16)	0.0078 (17)

### Geometric parameters (Å, º)

**Table:**

S1A—C1A	1.698 (3)	C6A—C7A	1.456 (3)
S1A—C4A	1.730 (2)	C4B—C3B	1.409 (3)
S1B—C1B	1.687 (3)	C3B—C2B	1.416 (3)
S1B—C4B	1.721 (2)	C3B—H3B	0.9300
O1B—C7B	1.383 (3)	C8A—C9A	1.475 (4)
O1B—C8B	1.389 (3)	C3A—C2A	1.397 (4)
O1A—C7A	1.385 (3)	C3A—H3A	0.9300
O1A—C8A	1.385 (3)	C6B—C7B	1.471 (3)
N1A—C8A	1.273 (3)	C8B—C9B	1.471 (4)
N1A—C6A	1.407 (3)	C2A—C1A	1.341 (4)
N1B—C8B	1.276 (3)	C2A—H2A	0.9300
N1B—C6B	1.405 (3)	C9B—H9B1	0.9600
C5B—C6B	1.332 (3)	C9B—H9B2	0.9600
C5B—C4B	1.438 (3)	C9B—H9B3	0.9600
C5B—H5B	0.9300	C1B—C2B	1.344 (4)
O2B—C7B	1.194 (3)	C1B—H1B	0.9300
C4A—C3A	1.369 (3)	C2B—H2B	0.9300
C4A—C5A	1.424 (3)	C1A—H1A	0.9300
O2A—C7A	1.193 (3)	C9A—H9A1	0.9600
C5A—C6A	1.340 (3)	C9A—H9A2	0.9600
C5A—H5A	0.9300	C9A—H9A3	0.9600
			
C1A—S1A—C4A	91.16 (12)	C5B—C6B—N1B	127.9 (2)
C1B—S1B—C4B	91.96 (13)	C5B—C6B—C7B	124.1 (2)
C7B—O1B—C8B	105.64 (19)	N1B—C6B—C7B	108.1 (2)
C7A—O1A—C8A	105.7 (2)	O2B—C7B—O1B	122.2 (2)
C8A—N1A—C6A	105.1 (2)	O2B—C7B—C6B	133.0 (3)
C8B—N1B—C6B	105.5 (2)	O1B—C7B—C6B	104.8 (2)
C6B—C5B—C4B	128.5 (2)	N1B—C8B—O1B	116.0 (2)
C6B—C5B—H5B	115.7	N1B—C8B—C9B	129.0 (3)
C4B—C5B—H5B	115.7	O1B—C8B—C9B	115.1 (2)
C3A—C4A—C5A	125.5 (2)	C1A—C2A—C3A	112.5 (3)
C3A—C4A—S1A	110.28 (18)	C1A—C2A—H2A	123.7
C5A—C4A—S1A	124.3 (2)	C3A—C2A—H2A	123.7
C6A—C5A—C4A	128.6 (2)	C8B—C9B—H9B1	109.5
C6A—C5A—H5A	115.7	C8B—C9B—H9B2	109.5
C4A—C5A—H5A	115.7	H9B1—C9B—H9B2	109.5
C5A—C6A—N1A	127.2 (2)	C8B—C9B—H9B3	109.5
C5A—C6A—C7A	124.3 (2)	H9B1—C9B—H9B3	109.5
N1A—C6A—C7A	108.5 (2)	H9B2—C9B—H9B3	109.5
C3B—C4B—C5B	125.3 (2)	C2B—C1B—S1B	113.0 (2)
C3B—C4B—S1B	110.85 (17)	C2B—C1B—H1B	123.5
C5B—C4B—S1B	123.87 (19)	S1B—C1B—H1B	123.5
O2A—C7A—O1A	122.1 (2)	C1B—C2B—C3B	113.6 (2)
O2A—C7A—C6A	133.2 (3)	C1B—C2B—H2B	123.2
O1A—C7A—C6A	104.7 (2)	C3B—C2B—H2B	123.2
C4B—C3B—C2B	110.6 (2)	C2A—C1A—S1A	112.9 (2)
C4B—C3B—H3B	124.7	C2A—C1A—H1A	123.6
C2B—C3B—H3B	124.7	S1A—C1A—H1A	123.6
N1A—C8A—O1A	116.0 (2)	C8A—C9A—H9A1	109.5
N1A—C8A—C9A	128.5 (3)	C8A—C9A—H9A2	109.5
O1A—C8A—C9A	115.5 (2)	H9A1—C9A—H9A2	109.5
C4A—C3A—C2A	113.1 (2)	C8A—C9A—H9A3	109.5
C4A—C3A—H3A	123.4	H9A1—C9A—H9A3	109.5
C2A—C3A—H3A	123.4	H9A2—C9A—H9A3	109.5
			
C1A—S1A—C4A—C3A	0.2 (2)	C7A—O1A—C8A—C9A	179.2 (2)
C1A—S1A—C4A—C5A	−179.6 (2)	C5A—C4A—C3A—C2A	179.7 (3)
C3A—C4A—C5A—C6A	178.7 (3)	S1A—C4A—C3A—C2A	0.0 (3)
S1A—C4A—C5A—C6A	−1.5 (4)	C4B—C5B—C6B—N1B	1.1 (4)
C4A—C5A—C6A—N1A	−1.2 (4)	C4B—C5B—C6B—C7B	−178.7 (2)
C4A—C5A—C6A—C7A	179.7 (3)	C8B—N1B—C6B—C5B	−179.8 (3)
C8A—N1A—C6A—C5A	−178.6 (3)	C8B—N1B—C6B—C7B	0.0 (3)
C8A—N1A—C6A—C7A	0.6 (3)	C8B—O1B—C7B—O2B	179.9 (3)
C6B—C5B—C4B—C3B	−176.6 (3)	C8B—O1B—C7B—C6B	−0.1 (3)
C6B—C5B—C4B—S1B	4.0 (4)	C5B—C6B—C7B—O2B	−0.2 (5)
C1B—S1B—C4B—C3B	0.1 (2)	N1B—C6B—C7B—O2B	180.0 (3)
C1B—S1B—C4B—C5B	179.6 (2)	C5B—C6B—C7B—O1B	179.9 (2)
C8A—O1A—C7A—O2A	−180.0 (3)	N1B—C6B—C7B—O1B	0.1 (3)
C8A—O1A—C7A—C6A	−0.1 (3)	C6B—N1B—C8B—O1B	−0.1 (3)
C5A—C6A—C7A—O2A	−1.2 (5)	C6B—N1B—C8B—C9B	179.1 (3)
N1A—C6A—C7A—O2A	179.5 (3)	C7B—O1B—C8B—N1B	0.2 (3)
C5A—C6A—C7A—O1A	178.9 (2)	C7B—O1B—C8B—C9B	−179.2 (2)
N1A—C6A—C7A—O1A	−0.3 (3)	C4A—C3A—C2A—C1A	−0.2 (4)
C5B—C4B—C3B—C2B	−179.9 (2)	C4B—S1B—C1B—C2B	0.2 (2)
S1B—C4B—C3B—C2B	−0.4 (3)	S1B—C1B—C2B—C3B	−0.4 (3)
C6A—N1A—C8A—O1A	−0.7 (3)	C4B—C3B—C2B—C1B	0.5 (3)
C6A—N1A—C8A—C9A	−179.2 (3)	C3A—C2A—C1A—S1A	0.4 (4)
C7A—O1A—C8A—N1A	0.5 (3)	C4A—S1A—C1A—C2A	−0.3 (2)

### Hydrogen-bond geometry (Å, º)

Cg1 is the centroid of the thiophene ring S1A/C1A–C4A.

**Table:**

*D*—H···*A*	*D*—H	H···*A*	*D*···*A*	*D*—H···*A*
C3*A*—H3*A*···O2*B*^i^	0.93	2.56	3.449 (3)	161
C3*B*—H3*B*···O2*A*^ii^	0.93	2.49	3.336 (3)	151
C9*B*—H9*B*2···*Cg*1^iii^	0.96	2.96	3.783 (4)	145

Symmetry codes: (i) −*x*+1, −*y*+2, −*z*+1; (ii) −*x*+1/2, *y*−1/2, −*z*+1/2; (iii) −*x*+1, −*y*+1, −*z*.
