# Supplementary figures and images for: Crystal structure of 2-methyl-4-[(thio­phen-2-yl)methyl­idene]-1,3-oxazol-5(4H)-one
Source: Acta Crystallogr E Crystallogr Commun. 2015 Jan 21;71(Pt 2):o123–4. doi: 10.1107/S2056989015000833 (PMC4384586; doi:10.1107/S2056989015000833)

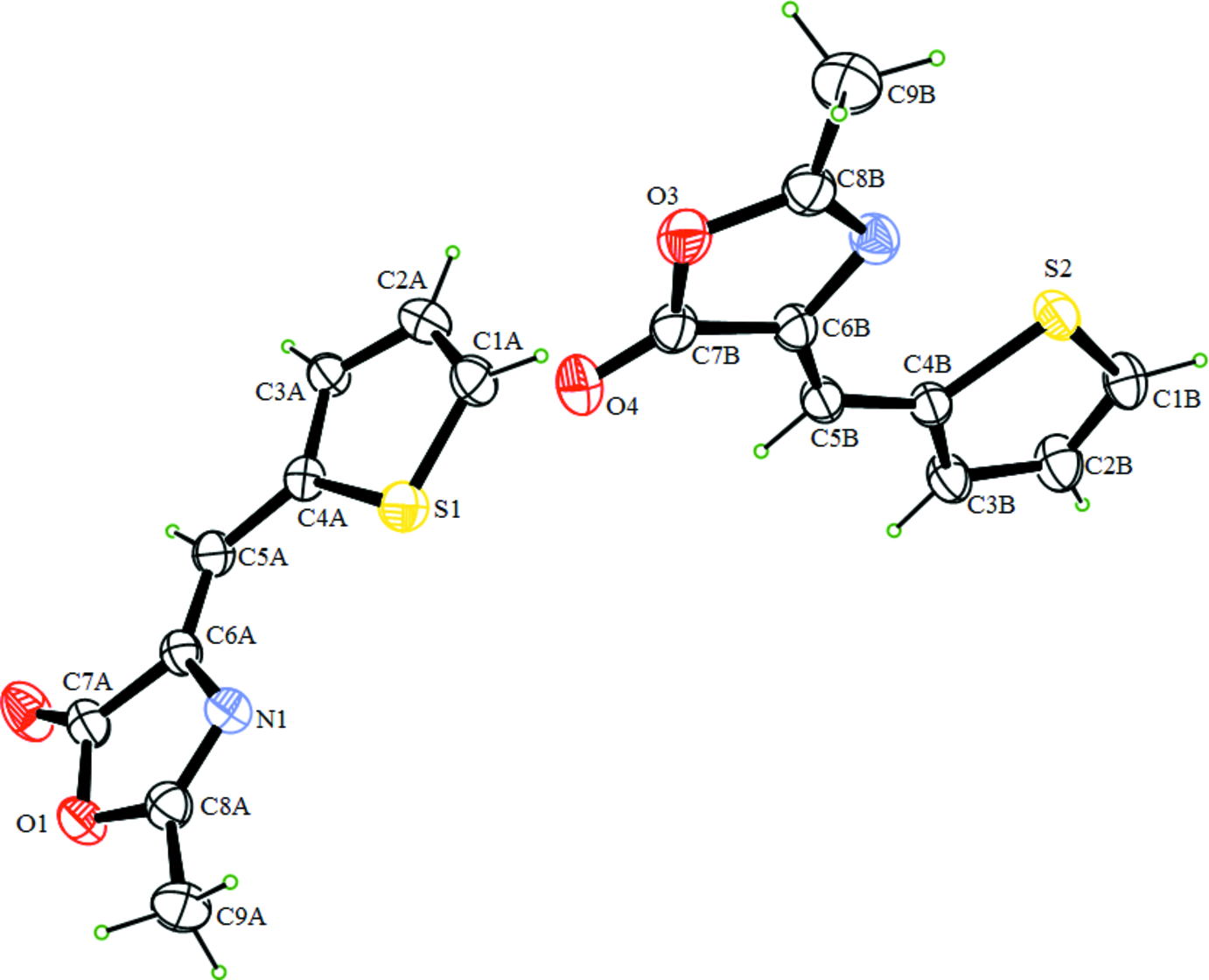

Supplement: Supplementary file 4 [file e-71-0o123-fig1.tif]

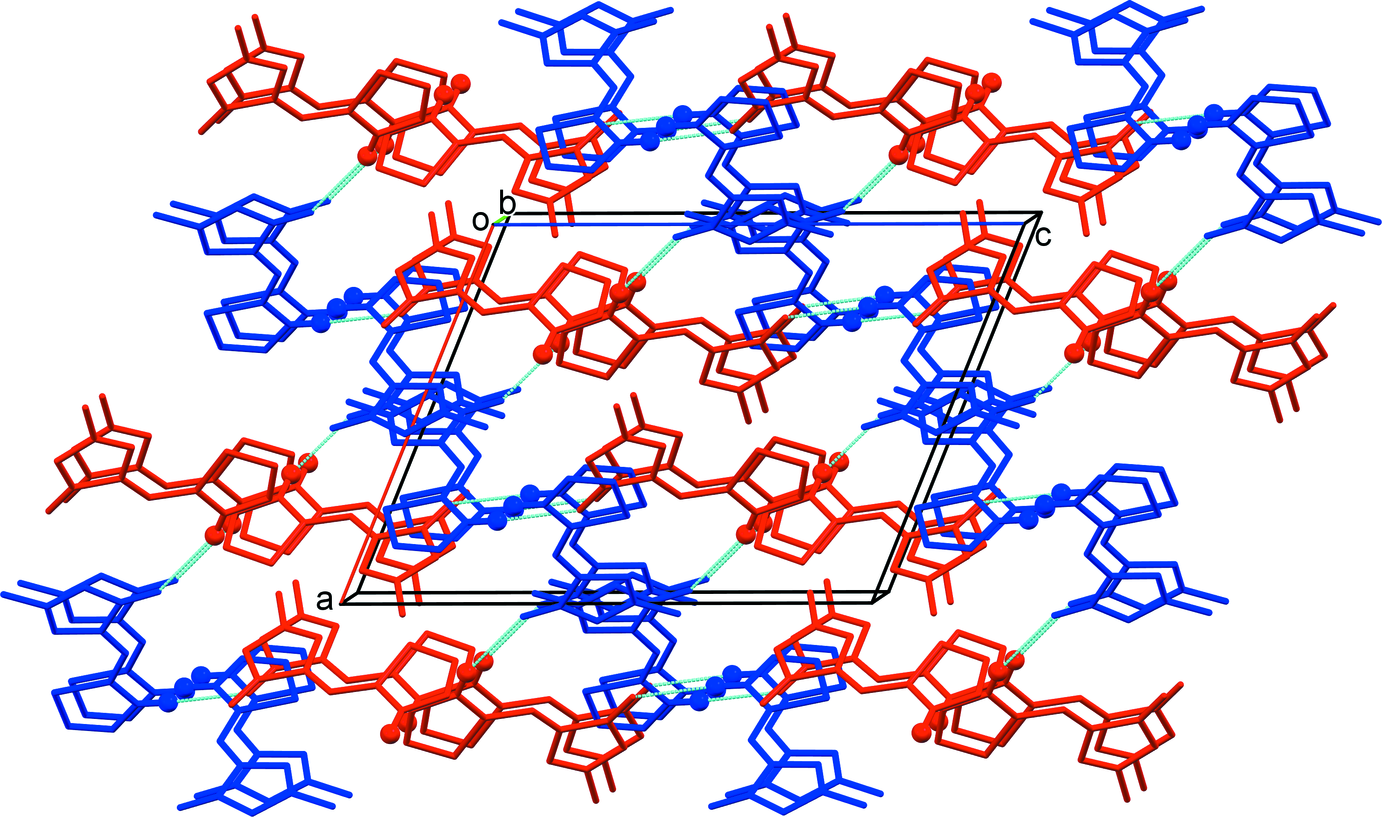

Supplement: Supplementary file 5 [file e-71-0o123-fig2.tif]
